# A set of domain-specific markers in the Arabidopsis embryo

**DOI:** 10.1007/s00497-015-0266-2

**Published:** 2015-07-28

**Authors:** Jos R. Wendrich, Barbara K. Möller, Borhan Uddin, Tatyana Radoeva, Annemarie S. Lokerse, Bert De Rybel, Dolf Weijers

**Affiliations:** Laboratory of Biochemistry, Wageningen University, Dreijenlaan 3, 6703 HA Wageningen, The Netherlands; Department of Plant Systems Biology, VIB, Technologiepark 927, 9052 Ghent, Belgium; Department of Plant Biotechnology and Bioinformatics, Ghent University, 9052 Ghent, Belgium; Department of Biochemistry and Molecular Biology, Jahangirnagar University, Dhaka, Savar, Bangladesh; Zentrum für Molekulare Biologie der Universität Heidelberg, Im Neuenheimer Feld 282, 69120 Heidelberg, Germany

**Keywords:** Arabidopsis, Embryo development, Tissue markers, Stem cells, *monopteros*

## Abstract

*****Key message***:**

**We describe a novel set of domain-specific markers that can be used in genetic studies, and we used two examples to show loss of stem cells in a*monopteros*background.**

**Abstract:**

Multicellular organisms can be defined by their ability to establish distinct cell identities, and it is therefore of critical importance to distinguish cell types. One step that leads to cell identity specification is activation of unique sets of transcripts. This property is often exploited in order to infer cell identity; the availability of good domain-specific marker lines is, however, poor in the Arabidopsis embryo. Here we describe a novel set of domain-specific marker lines that can be used in Arabidopsis (embryo) research. Based on transcriptomic data, we selected 12 genes for expression analysis, and according to the observed expression domain during embryogenesis, we divided them into four categories (1—ground tissue; 2—root stem cell; 3—shoot apical meristem; 4—post-embryonic). We additionally show the use of two markers from the “stem cell” category in a genetic study, where we use the absence of the markers to infer developmental defects in the *monopteros* mutant background. Finally, in order to judge whether the established marker lines also play a role in normal development, we generated loss-of-function resources. None of the analyzed T-DNA insertion, artificial microRNA, or misexpression lines showed any apparent phenotypic difference from wild type, indicating that these genes are not nonredundantly required for development, but also suggesting that marker activation can be considered an output of the patterning process. This set of domain-specific marker lines is therefore a valuable addition to the currently available markers and will help to move toward a generic set of tissue identity markers.

**Electronic supplementary material:**

The online version of this article (doi:10.1007/s00497-015-0266-2) contains supplementary material, which is available to authorized users.

## Introduction

The establishment of distinct cell identities is a central property of multicellular organisms, and it is therefore of critical importance to distinguish cell types. Typically, cell identity specification involves the activation (or repression) of a unique set of transcripts, followed by the accumulation of proteins and ultimately by cell differentiation events. Cell identity can be inferred at any of these steps, by transcriptional output, molecular composition, or morphology and shape. One of the most widely used markers for intrinsic cell identity is transcriptional output, either by in situ mRNA hybridization or using promoters of cell-type-specific genes driving a reporter protein that can be visualized using, e.g., fluorescence or histological coloring in transgenic plants. Gene expression markers are very powerful, as their activation is one of the first events in cell specification. However, an intrinsic drawback of using gene expression reporters is that each gene is regulated by an intricate network, and even if a gene’s pattern reflects a cell identity, it does not define it. Therefore, one would ideally combine several cell identity markers to infer identity.

The Arabidopsis life cycle starts when an egg cell is fertilized and embryogenesis is initiated. During the process of embryogenesis, cells in the embryo undergo several rounds of division and specification events that establish tissue and cell types de novo. It is these de novo specification events that make embryogenesis into an excellent model for studying several developmental processes, as they can teach us how cell and tissue identity is being established (Wendrich and Weijers 2013). In addition, all these events happen in a relatively short time span and occur in a very strict and orderly fashion, which makes it easier to infer underlying processes when development is disturbed. One property of Arabidopsis embryogenesis as a model system is still lagging however, the availability of gene expression markers. Some markers have already been well established (e.g., Aida et al. 2004; De Rybel et al. 2013; Haecker et al. 2004), but the amount of markers available is very limited and surely does not result in a saturation of the different possible regions and cell types.

Here we describe the establishment of a novel set of domain-specific markers in the Arabidopsis embryo. Based on both published (Le et al. 2010; Brady et al. 2007) and our own unpublished transcriptomic data from embryonic and root tissues, we have selected 12 genes for expression analysis. Here we report the expression domains of all of them in several stages of embryonic development and in the post-embryonic primary root, as reported by their putative promoters. In addition, we show their usefulness as marker lines in genetic studies.

## Methods

### Plant material and growth conditions

T-DNA insertion lines WiscDsLox466B7/*spt*-11 and WiscDsLox386E06/*spt*-12 were described in Ichihashi et al. (2010) and obtained from the Nottingham Arabidopsis Stock Centre (NASC), along with SALK_103775, FLAG_399C07, SAIL_318_C07, and SALK_068811. Insertions were genotyped using primers listed in Supplementary Table 1.

All Arabidopsis seeds were surface-sterilized and grown on ½ MS plates either with or without selective antibiotics in standard long-day (16:8-h light/dark) growth conditions at 22 °C in a growth room. Fourteen-day-old seedlings were transferred to soil and grown further in the same conditions.

### Cloning and plant transformation

Up to 5 kb upstream of the start codon was cloned into the pPLV04 or pPLV04_v2 vector using ligation-independent cloning (De Rybel et al. 2011; Wendrich et al. 2015) and primers described in Supplementary Table 1.

Knockdown lines using artificial microRNA (amiRNA) were constructed as described by Schwab et al. (2006) and complete coding sequences were amplified, using primers defined in Supplementary Table 1. Constructed amiRNAs and coding sequences were cloned into the pPLV028 vector (De Rybel et al. 2011) for broad embryonic expression under the RPS5A promoter (Weijers et al. 2001).

All constructs were confirmed by sequencing and transformed into Col-0 wild type or *mp*-B4149 heterozygous (Weijers et al. 2006) Arabidopsis plants by simplified floral dipping (De Rybel et al. 2011).

### Microscopy

#### Differential interference contrast (DIC)

DIC microscopy was performed on isolated ovules or 6-day-old seedling roots as described previously (Llavata-Peris et al. 2013). Briefly, samples were cleared in a chloral hydrate solution (chloral hydrate, water and glycerol [8:3:1]) and observed with a Leica DMR microscope equipped with DIC optics.

#### Confocal laser scanning microscopy (CLSM)

CLSM was performed as described previously (Llavata-Peris et al. 2013) with some modifications. Briefly, ovules were isolated and fixed in a 4 % paraformaldehyde/5 % glycerol in PBS solution containing 1.5 % SCRI Renaissance Stain 2200 (R2200; Renaissance Chemicals, UK) for counterstaining of embryos. Embryos were popped out of the ovules, and R2200 and GFP were visualized by excitation at 405 and 488 nm and detection between 430–470 and 500–535 nm, respectively. For imaging of roots, 6-day-old seedlings were submerged in water containing 1.5 % FM4-64 (Invitrogen) for 1–2 min and GFP and FM4-64 were visualized by excitation at 488 nm and detection between 500–535 and 630–700 nm, respectively. All CLSM was performed on a Leica SP5 system equipped with Hybrid Detector.

## Results and discussion

### Selection of genes and establishment of marker lines

Based on both publically available (Le et al. 2010; Brady et al. 2007; Winter et al. 2007; Supplementary Figure 1) and our own unpublished transcriptome data, collected from misregulation of known factors involved in development, we selected twelve genes (Table 1) that were expected to show local expression in the early embryo (Supplementary Figure 2). In order to utilize the expression of the selected genes as markers for early embryo development, we cloned the putative promoters, up to 5 kb upstream of the start codon, to drive the expression of a nuclear localized triple green fluorescent protein (n3GFP) and transformed these into Arabidopsis. The n3GFP has high fluorescence intensity and is concentrated in the cell nucleus, which makes it a good tool for expression analyses in Arabidopsis embryos (Takada and Jürgens 2007; Rademacher et al. 2011). More than three independent transgenic lines were analyzed for each of the 12 constructs, and we here report the representative patterns observed in the majority of lines. Since gene expression is not always accurately represented by promoter fragments, but may also depend on sequences downstream of the transcriptional start site, we do not consider these lines as representatives of gene expression per se. Rather, we consider these as tools that can act as molecular markers for cell or domain identity, irrespective of gene function. Consequently, although the majority of the lines showed an overlapping expression domain compared to the published transcriptomics data (Supplementary Figures 1 and 2), not all lines show a similar expression pattern as was expected. This shows there can be differences between putative promoter activity and detectable transcripts.

**Table 1:** Overview of observed expression patterns in embryo and constructed and tested knockdown and misexpression lines

Category	AGI	Gene description	Observed expression	Insertion lines^b^	amiRNA^b^	Misexpression^b^
Ground tissue	At1g05710	Basic helix-loop-helix domain-containing protein	Starting at globular stage expression in ground tissue cells of future root and cotyledons	FLAG_399C07	×	×
	At2g31730	Basic helix-loop-helix domain-containing protein	Starting before globular stage expression in suspensor, later in ground tissue cells of future root and cotyledons	SAIL_318_C07 SALK_068811	×	×
Stem cell	At2g03830	Unknown protein root meristem growth factor 8 (RGF8)	Starting before globular stage, strong expression in suspensor, later expends to basal embryo	NA	×	×
	At3g19380	U-box domain-containing protein 25	Starting at globular stage, expression in basal embryo	NA	NA	NA
	At4g36930	Transcription factor SPATULA	Starting at globular stage, expression in basal embryo	WiscDsLox466B7 (*spt*-11) WiscDsLox386E06 *(spt*-12)^a^	×	×
	At5g60810	Root meristem growth factor 1	Starting at globular stage, expression in suspensor and later in basal embryo		×	×
Shoot apical meristem	At5g67110	Transcription factor ALC	Starting at heart stage, expression in outer layer of SAM region	SALK_103775	×	×
Post-embryonic	At1g26945	Basic helix-loop-helix protein KIDARI	Expression in first few xylem cells	NA	NA	NA
	At2g17070	Hypothetical protein; Arabidopsis protein of unknown function (DUF241)	Expression in lateral root cap cells	NA	NA	NA
	At3g04430	NAC domain-containing protein 49	Ubiquitous expression in root meristem	NA	NA	NA
	At3g23880	F-box/kelch-repeat protein	Ubiquitous expression in root meristem	NA	NA	NA
	At5g62330	Hypothetical protein	Expression in lateral root cap cells	NA	NA	NA

*NA* not analyzed

^a^Ichihashi et al. (2010)

^b^≥100 embryos were observed

Using confocal microscopy, we found that seven of the markers showed expression of n3GFP during embryo development and the remaining five were expressed only later during post-embryonic root development (Supplementary Figure 3; Table 1). Based on these findings, we grouped the remaining seven genes into three different categories, depending on the observed expression pattern in the embryo (1—ground tissue; 2—root stem cell; 3—shoot apical meristem). Each of the reported patterns is robust, as embryo-to-embryo variation is minimal (Supplementary Figure 4).

### Ground tissue lines

**Fig. 1:**
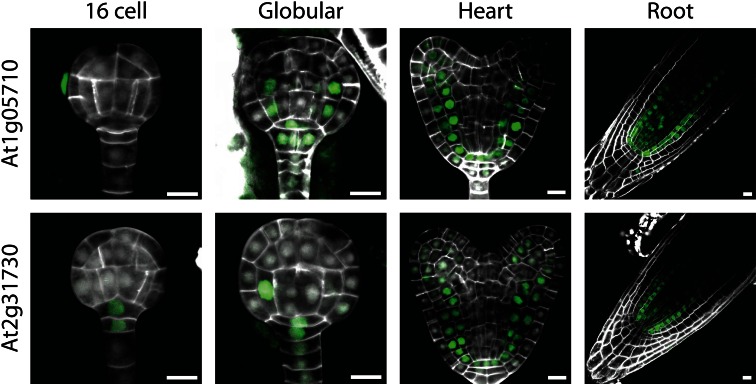
Expression of genes in the ground tissue category, shown in globular and heart-stage embryos and in post-embryonic root. Expression was observed in all types of ground tissue cells, i.e., both root and shoot derived. *Scale bars* 10 µm

The first category consisted of two genes (At1g05710 and At2g31730), encoding two basic helix-loop-helix (bHLH) transcription factors, that were found expressed (in the case of At2g31730) as early as the 16-cell stage in the suspensor and future hypophysis and later expanded their expression to all cells of the ground tissue (Fig. 1). Interestingly, this group of genes is expressed not only in the ground tissue cells of the future root, but also in the ground tissue cells of the hypocotyl and developing cotyledons, i.e., mesophyll precursor cells (Fig. 1). In the post-embryonic root, these genes showed a similar expression domain as found in embryos, except that weak expression in vascular cells could also be detected (Fig. 1). To our knowledge, this is the first demonstration of a “pan-ground tissue” pattern that not only marks endodermis and cortex, but also the mesophyll, at least within the embryo. This is striking because the ground tissue of root and hypocotyl has a different origin compared to that of the cotyledons. The root and hypocotyl ground tissue derives from four precursor cells in the lower half of the pro-embryo that form after periclinal division of the inner cells at 16-cell stage (Yoshida et al. 2014). Indeed, expression of At2g31730 is already detected in this precursor cell (Fig. 1). The cotyledon ground tissue (mesophyll) is instead derived from the upper half of the pro-embryo, in which the ground tissue lineage cannot as easily be predicted. Indeed, no expression of these two ground tissue markers can be detected at globular stage in the upper half of the embryo (Fig. 1). Thus, despite having a different ontogeny, the entire ground tissue appears to share expression of at least these two genes. For this reason, we believe that these reporters can serve as generic markers for developing ground tissue, which are specific during heart-stage embryogenesis.

### “Root stem cell” lines

**Fig. 2:**
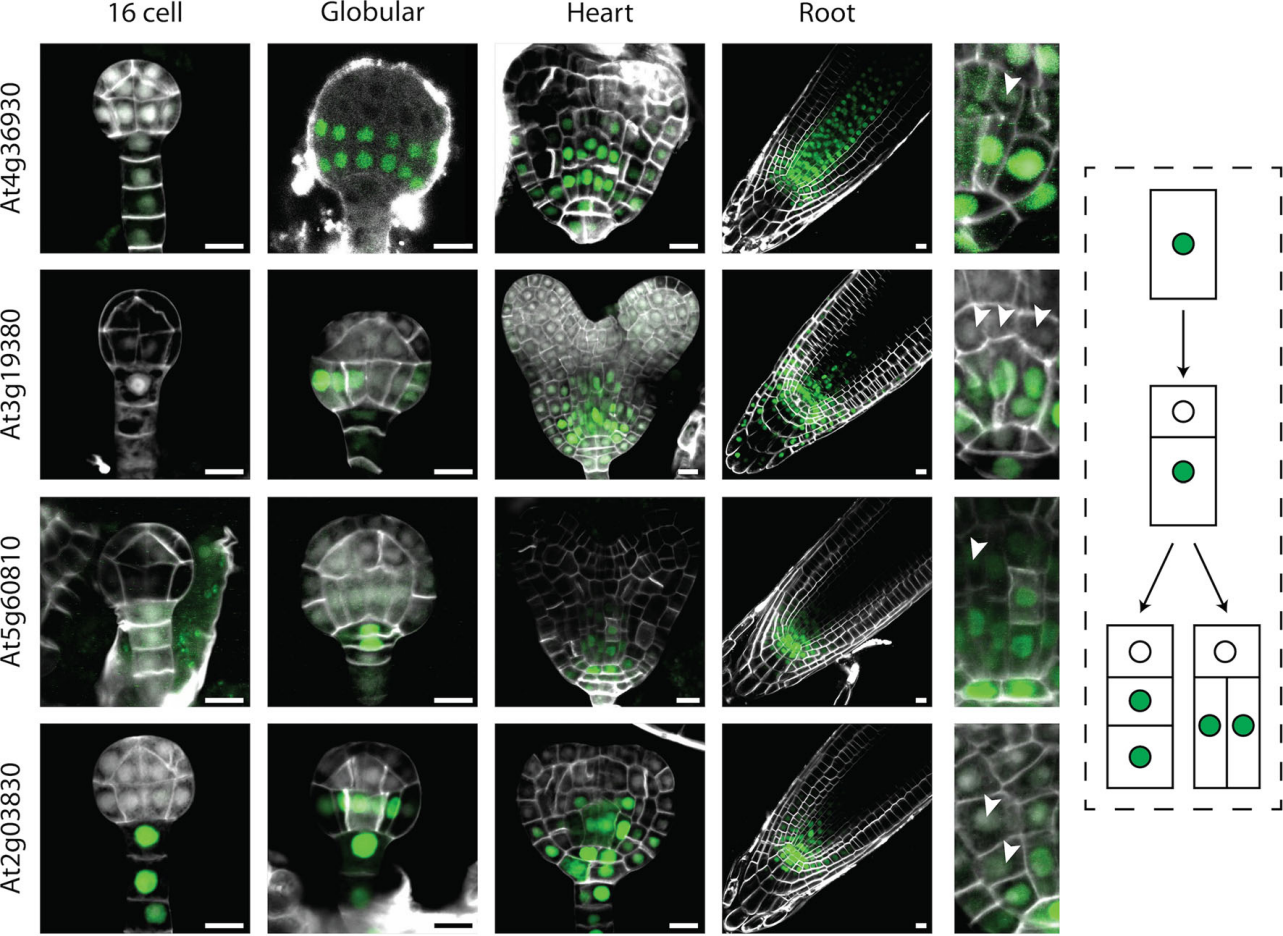
Expression of genes is the “root stem cell” category, shown in 16-cell, globular, and heart-stage embryos and in post-embryonic root. Expression was observed in the basal part of the embryo, coinciding with the area of stem cells. *Arrowheads* in the *right* images indicate loss of expression in daughter cells further displaced from the QC, as is also depicted schematically on the *right*. *Scale bars* 10 µm

The second category consisted of four genes (At2g03830, At3g19380, At4g36930, and At5g60810), encoding one bHLH transcription factor (SPATULA [SPT]; At4g36930; Heisler et al. 2001), one PLANT U-BOX domain-containing protein (At3g19380), and two peptides (ROOT GROWTH FACTOR1 [RGF1]: At5g60810; RGF8: At2g03830; Matsuzaki et al. 2010), that showed similar expression patterns, marking the lower half of the globular embryos, and being restricted to a smaller domain of cells surrounding the lens-shaped cell. As the lens-shaped cell is the precursor to the root quiescent center (QC; Scheres et al. 1994), the cells surrounding it are considered stem cells (Bennett and Scheres 2010). In post-embryonic roots, expression of all these four genes was found in a zone surrounding the QC, with highest expression directly adjacent to the QC. As the expression of these genes did not appear to be correlated with zones of cell division in general (Burssens et al. 2000; Weijers et al. 2001), nor was there any tissue- specificity, we interpret these genes to mark a property that is related to the stem cells in the root. The moment and location of gene activation of these genes are not identical, nor are the patterns in the root. Nonetheless, these genes share a common pattern at heart stage and collectively mark the youngest cells in the root meristem. Thus, we refer to these genes as potential stem cell markers. An important consideration is that all genes show striking expression dynamics in the basal embryo pole. Upon division, expression is retained in the cells closest to the lens-shaped cell, while it is lost from the daughter that is displaced from the lens-shaped cell (Fig. 2). This, to our interpretation, resembles a self-renewal division known for stem cells (Bennett and Scheres 2010; Wendrich and Weijers 2013). This implies that genes from this group could be used as markers for the stem cell region during both embryo and root development.

### Shoot apical meristem line

**Fig. 3:**
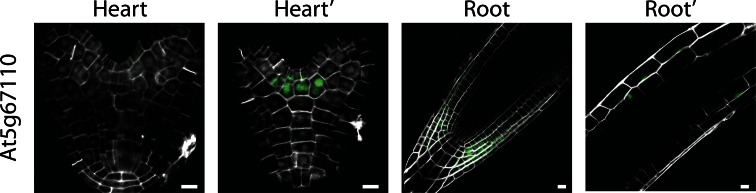
Expression of gene in the apical meristem category, shown in heart-stage embryos and in post-embryonic root. Expression was observed in the outermost cell layer at the cotyledon boundary. *Scale bars* 10 µm

The third category was defined by a single gene, encoding a bHLH transcription factor (ALCATRAZ; At5g67110; Rajani and Sundaresan 2001) that did not show expression during the earliest steps of embryo development, but whose activity was observed starting around the heart stage of development in the outermost layer of the epidermis (Fig. 3). This gene, At5g67110, was specifically expressed in the boundaries between the two cotyledon primordial, but not in the shoot apical meristem. As such, the pattern resembled that of the CUP-SHAPED COTYLEDON (CUC) genes (Aida et al. 1999; Hibara et al. 2003; Vroemen et al. 2003). In the post-embryonic root, expression was observed in the epidermal and lateral root cap layers as well as in differentiating cortex cells (Fig. 3). To our knowledge, there is no obvious fate or property common to these three domains (cotyledon boundary, root cap, and mature cortex). Hence, this reporter can be used as cotyledon boundary marker in the embryo context, but it should be noted that it does not define this cell type.

### Genetic regulation of marker expression

**Fig. 4:**
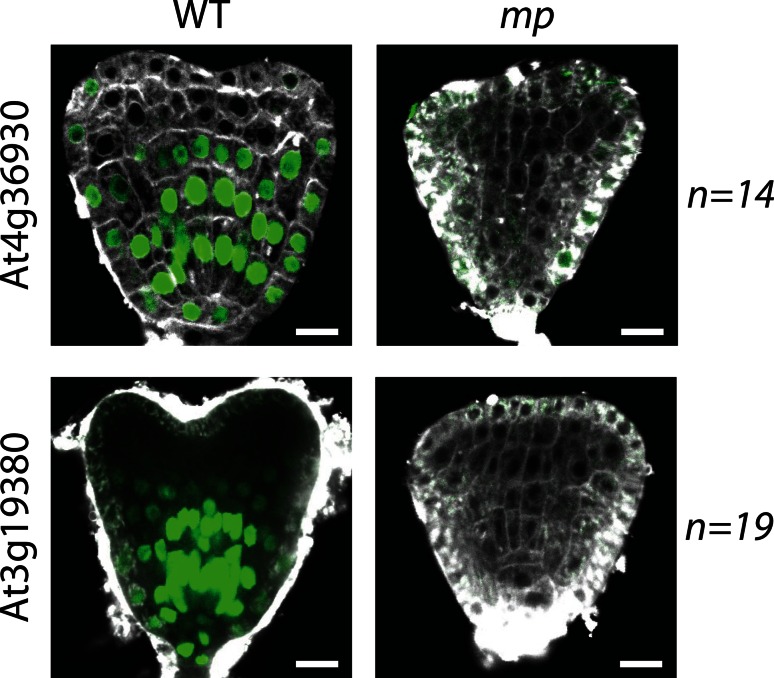
Expression of two genes from the “root stem cell” category in wild type and in *mp* background, shown in heart-stage embryos. Expression was observed as described before in wild type, though expression was completely lost in the *mp* mutant, indicating stem cell specification defects in *mp*. *Scale bars* 10 µm

The purpose of gene expression markers is not only to inform about molecular differences between cells and domains in the wild type, but importantly also to help interpret mutant defects. We assessed the usefulness of several of the newly established marker lines in genetic studies. For this purpose, we used the *monopteros* mutant (*mp*; Berleth and Jurgens 1993; Hardtke and Berleth 1998; Weijers et al. 2006), which displays a characteristic rootless phenotype. During embryogenesis, the *mp* mutant can be identified based on aberrant cell division planes in the hypophysis and the adjacent cells in the pro-embryo (Berleth and Jurgens 1993; Hardtke and Berleth 1998). However, a largely unanswered question is what processes are actually disturbed in the mutant. Individual genes have been shown to be regulated by MP and consequently are downregulated in the mutant (Schlereth et al. 2010). Yet, these genes are not only expressed in specific patterns in the wild type, but also themselves required for normal development (De Rybel et al. 2013). Hence, to better understand the developmental role of the MP protein, it will be helpful to analyze the activity of other markers in the mutant. By transformation into a heterozygous *mp* background, we were able to show that the expression of the two “stem cell” markers At4g36930 and At3g19380 was completely lost in the *mp* mutant (Fig. 4). This finding indicates that (1) the markers are under genetic control by a pathway that involves the MP protein, (2) co-expression of these two genes in wild type reflects co-regulation by the same pathway, and (3) MP may control stem cell specification in the Arabidopsis embryo. Loss of *PLT1* and *PLT2* expression in the *mp*/*arf5 nph4*/*arf7* double mutant (Aida et al. 2002) had previously suggested that MP is required for aspects of meristem formation. We believe that the loss of expression of these two entirely unrelated genes in the *mp* mutant lends support to the idea that *mp* has a significant stem cell specification defect. Especially the latter suggestion could not previously be made due to the absence of markers.

### Gene function of marker lines

Finally, in order to judge whether the marker lines generated here are only activated as part of the cell/domain specification process, or whether they play an important role in the specification process, we generated loss-of-function resources. We analyzed several T-DNA insertion lines and additionally generated artificial microRNA and misexpression lines (Table 1). No apparent phenotypic difference compared to wild type was found in any of the analyzed lines, indicating that disruption of expression of these genes does not affect development. While this result demonstrates that none of the genes reflected by the markers is nonredundantly required for normal development, it also suggests that marker activation can be considered a true output of the patterning process. This renders these lines useful proxies for determining cell/domain identity during embryo development.

## Conclusion

In an effort to increase the number of useful cell/domain markers, we have generated a set of marker lines that can be used for (genetic) studies in Arabidopsis embryos. We show four different categories of markers, based on their expression in the Arabidopsis embryo: 1—ground tissue; 2—stem cell; 3—shoot apical meristem; 4—post-embryonic. While some of these mark previously described domains (stem cell region, cotyledon boundaries), others mark a novel domain. Good examples are the two ground tissue markers that can be considered pan-ground-tissue markers. These do not only mark both endodermis and cortex, but are also active in the entire ground tissue domain of future cotyledon, hypocotyl, and root. The activity of these markers identifies a convergent molecular signature in ground tissue with different cellular ontogeny and can help to better understand the mechanisms of ground tissue specification.

We used two of the stem cell markers to show that the rootless phenotype displayed by the *mp* mutant is accompanied by a lack of activation of “stem cell” markers in the basal region of the embryo, which is likely a consequence of the aberrant divisions in early stages of development. Analysis of several lines including multiple strategies for expression disruption showed no phenotypic alterations during embryo development, supporting the usefulness of these marker lines as output reporters in a developmental context. This set of marker lines is a valuable addition to the currently available set of markers, as it will help to move away from regulation on single genes toward a more generic set of tissue identity markers.

### **Author contribution statement**

JRW, BKM, BU, TR, AL, and BDR constructed and analyzed promoter reporter lines, JRW and BU constructed and analyzed misexpression and amiRNA lines and analyzed T-DNA insertion lines, BKM and JRW analyzed expression in mutant background, DW conceived the research, and JRW and DW wrote the paper with input from all other authors.

## Electronic supplementary material

Supplementary Figure 1 Gene expression visualization of selected genes in eFP browser, based on cell-type-specific transcriptomic data on Arabidopsis roots (Brady et al. 2007; Winter et al. 2007) (PDF 811 kb)

Supplementary Figure 2 Gene expression visualization of selected genes in eFP browser, based on transcriptomic data on different parts of developing seeds (Le et al. 2010; Winter et al. 2007) (PDF 129 kb)

Supplementary Figure 3 Expression of genes in the post-embryonic category, shown in post-embryonic root. Expression for two genes was observed in the lateral root cap, for one in early xylem cells and for two others ubiquitously in the root meristem. Scale bars = 10 µm (PNG 3180 kb)

Supplementary Figure 4 Expression of all embryo-expressing lines in wild-type heart-stage embryos, shown in different individuals. This indicates the robustness of observed expression patterns. Scale bars = 10 µm (PNG 4563 kb)

Supplementary Table 1 List of oligonucleotides used in this study (XLS 43 kb)
