# Supplementary figures and images for: A set of domain-specific markers in the Arabidopsis embryo
Source: Plant Reprod. 2015 Jul 28;28(3-4):153–60. doi: 10.1007/s00497-015-0266-2 (PMC4623083; doi:10.1007/s00497-015-0266-2)

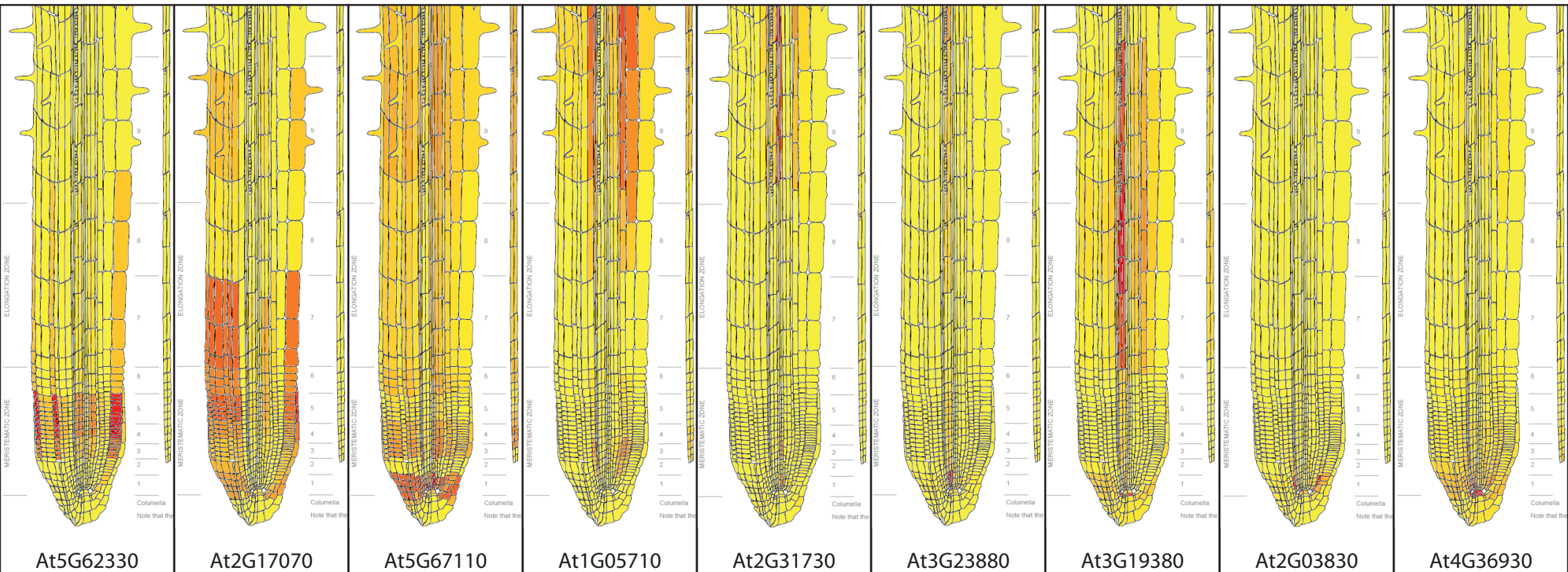

Supplement: Supplementary file 1 — Gene expression visualization of selected genes in eFP browser, based on cell-type-specific transcriptomic data on Arabidopsis roots (Brady et al. 2007; Winter et al. 2007) (PDF 811 kb) [file 497_2015_266_MOESM1_ESM.pdf]

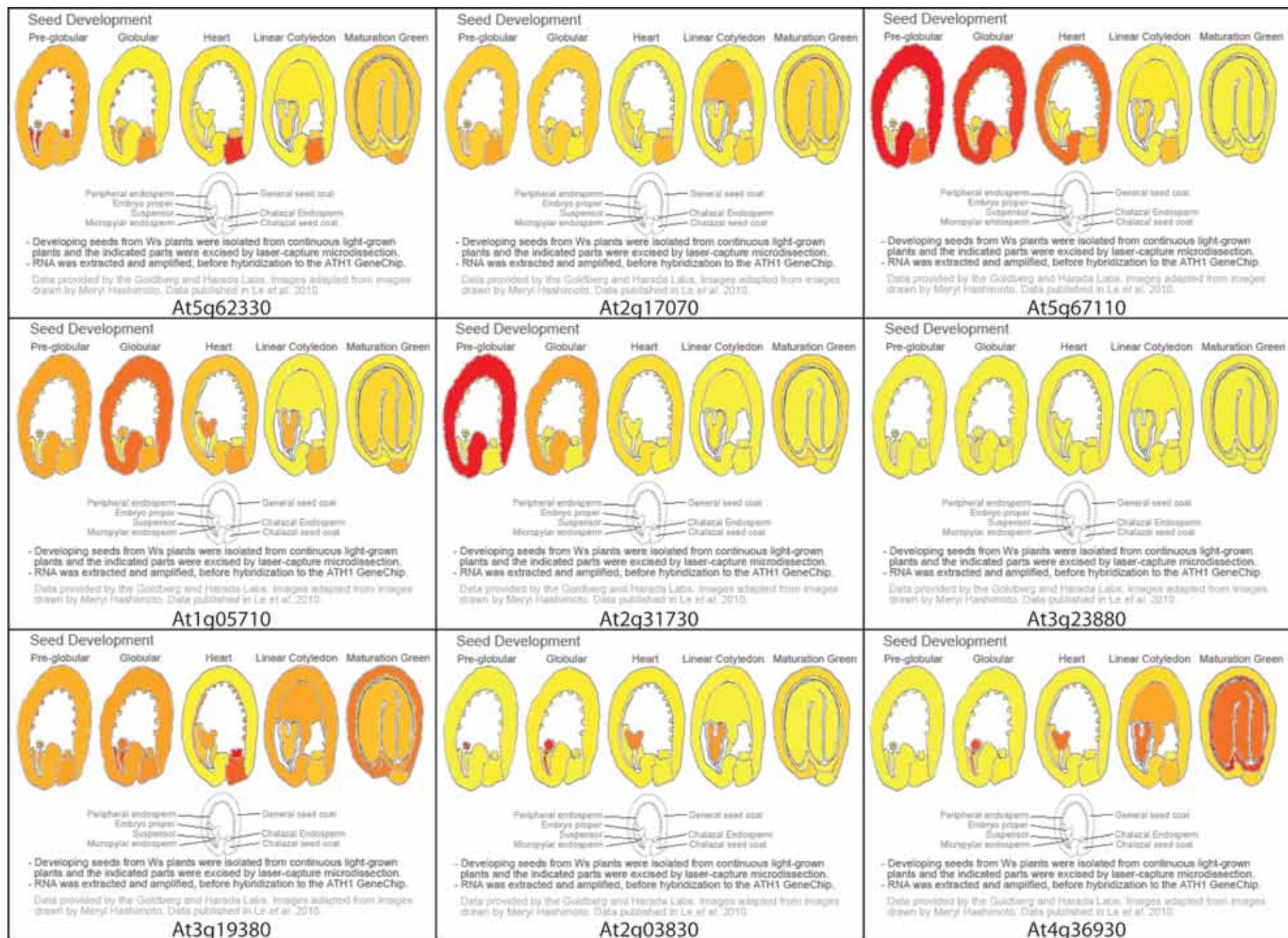

SupFigure 2

Supplement: Supplementary file 2 — Gene expression visualization of selected genes in eFP browser, based on transcriptomic data on different parts of developing seeds (Le et al. 2010; Winter et al. 2007) (PDF 129 kb) [file 497_2015_266_MOESM2_ESM.pdf]

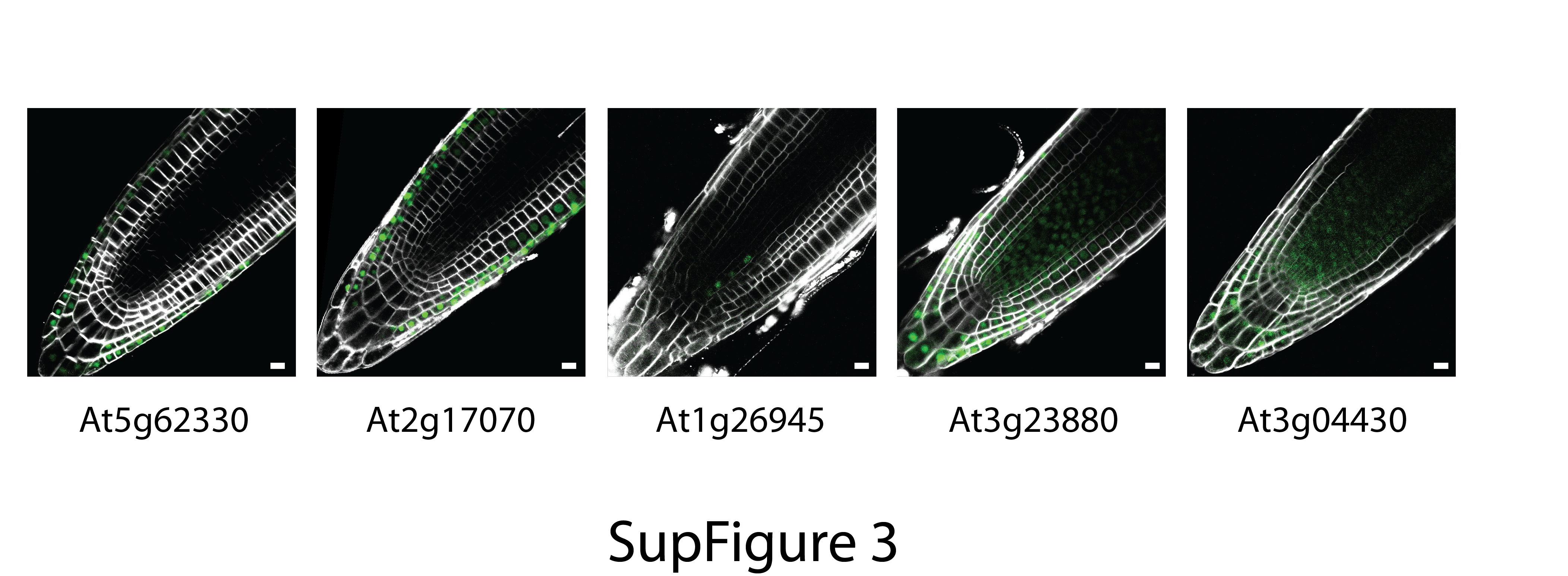

Supplement: Supplementary file 3 — Expression of genes in the post-embryonic category, shown in post-embryonic root. Expression for two genes was observed in the lateral root cap, for one in early xylem cells and for two others ubiquitously in the root meristem. Scale bars = 10 µm (PNG 3180 kb) [file 497_2015_266_MOESM3_ESM.png]

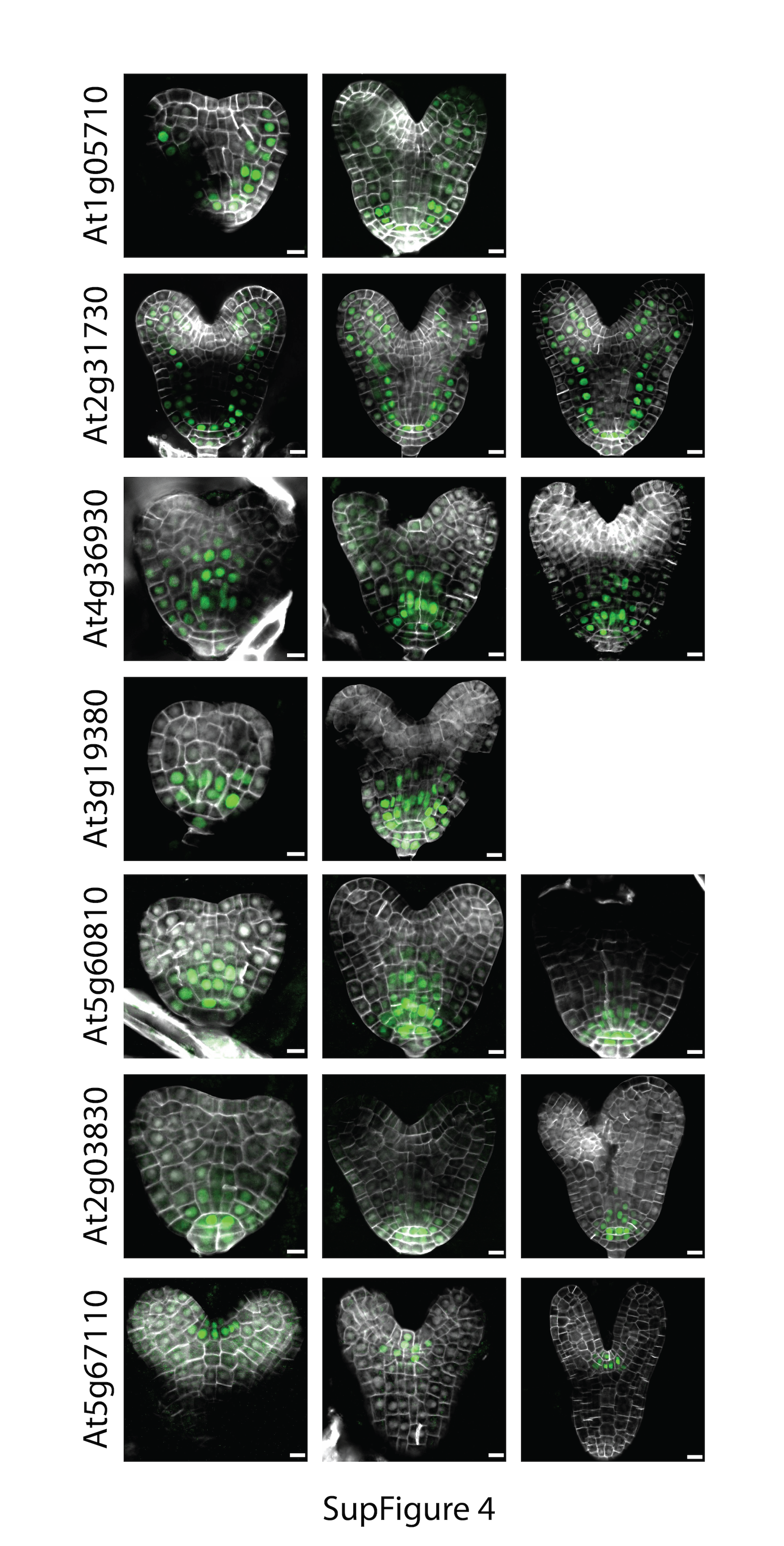

Supplement: Supplementary file 4 — Expression of all embryo-expressing lines in wild-type heart-stage embryos, shown in different individuals. This indicates the robustness of observed expression patterns. Scale bars = 10 µm (PNG 4563 kb) [file 497_2015_266_MOESM4_ESM.png]
